# Health, educational and employment outcomes among children treated for a skin disorder: Scotland-wide retrospective record linkage cohort study of 766,244 children

**DOI:** 10.1371/journal.pone.0243383

**Published:** 2020-12-11

**Authors:** Michael Fleming, James S. McLay, David Clark, Albert King, Daniel F. Mackay, Jill P. Pell

**Affiliations:** 1 Institute of Health and Wellbeing, University of Glasgow, Glasgow, United Kingdom; 2 Department of Child Health, University of Aberdeen, Aberdeen, United Kingdom; 3 Public Health Scotland, Edinburgh, United Kingdom; 4 ScotXed, Scottish Government, Edinburgh, United Kingdom; Sciensano, BELGIUM

## Abstract

**Background:**

To compare health, educational and employment outcomes of schoolchildren receiving medication for a skin disorder with peers.

**Methods:**

This retrospective population cohort study linked eight Scotland-wide databases, covering dispensed prescriptions, hospital admissions, maternity records, death certificates, annual pupil census, school examinations, school absences/exclusions and unemployment to investigate educational (absence, exclusion, special educational need, academic attainment), employment, and health (admissions and mortality) outcomes of 766,244 children attending local authority run primary, secondary and special schools in Scotland between 2009 and 2013.

**Results:**

After adjusting for sociodemographic and maternity confounders the 130,087 (17.0%) children treated for a skin disorder had increased hospitalisation, particularly within one year of commencing treatment (IRR 1.38, 95% CI 1.35–1.41, p<0.001) and mortality (HR 1.50, 95% CI 1.18–1.90, p<0.001). They had greater special educational need (OR 1.19, 95% CI 1.17–1.21, p<0.001) and more frequent absences from school (IRR 1.07, 95% CI 1.06–1.08, p<0.001) but did not exhibit poorer exam attainment or increased post-school unemployment. The associations remained after further adjustment for comorbid chronic conditions.

**Conclusions:**

Despite increased hospitalisation, school absenteeism, and special educational need, children treated for a skin disorder did not have poorer exam attainment or employment outcomes. Whilst findings relating to educational and employment outcomes are reassuring, the association with increased risk of mortality is alarming and merits further investigation.

## Background

Skin disorders including psoriasis and eczema, also referred to as atopic dermatitis, are the fourth leading cause of disability worldwide [1]. These disorders have an impact on quality of life [2–6] and create a burden on healthcare resources [4–6]. The annual cost of eczema and psoriasis in the USA have been estimated at $5.3billion [7] and as much as $63.2billion [8] respectively and it has been reported that skin diseases globally contributed 1.8% (18th leading contributor) and 1.4% (22nd leading contributor) to disability-adjusted life years (DALYs) in 2013 and 2016 respectively; eczema and psoriasis contributing 0.4% and 0.2% respectively in the former report [1,9]. Skin disorders are more common in children than adults. Worldwide, the prevalence of eczema has been reported as 17–20% in children compared with 1–3% in adults [10,11]. Childhood prevalence varies greatly between countries [12,13], ranging from 0.2% in China to 24.6% in Columbia. Psoriasis is a less common condition in children but still affects around 0.5% [14]. Children who have a skin disorder often suffer from additional medical conditions. Studies have reported increased risk of asthma, allergic rhinitis, hay fever and autoimmune disorders among children with eczema [15–18] and a higher prevalence of obesity, diabetes, cardiovascular disorders, rheumatoid arthritis, and Crohn’s disease among children with psoriasis [14,19–21]. Eczema and psoriasis have also been linked with a number of psychiatric disorders and mental health problems including depression and anxiety [22–28]. In addition to depression, childhood eczema has been associated with comorbid attention deficit hyperactivity disorder (ADHD) [29–31] and suicidal ideation/behaviour [32–34]. Adults with skin disorders reportedly suffer from increased mortality [35,36] and, whilst studies investigating hospitalisation and mortality in children and adolescents are rare, increased psychiatric hospitalisation [37] and mortality [38] among children with psoriasis have been reported.

Given that these disorders not only impact on physical and mental health but also affect sleep and daily function [39], it is plausible that having a skin disorder may adversely affect educational attainment and employment. Increased absence from school and work [39] and perceived school difficulty among students [40] have been reported, however a recent systematic review of educational and employment outcomes among children with eczema identified only one eligible study [41]. The study reported no association between eczema and performance in a secondary school entrance exam undertaken at 11years of age but did not have information on subsequent school academic attainment or employment after leaving school [42]. A subsequent national study across Sweden found no associations between atopic dermatitis and lower cognitive function or lower academic attainment but did not investigate other educational or health outcomes. Further, it only studied men aged 17–20 between 1969 and 1976 who possibly had more severe atopic dermatitis, limiting generalizability [43]. This study addresses a significant gap in the literature by investigating school attendance, educational attainment, and employment, as well as hospitalisations and mortality, in a single unselected country-wide cohort of schoolchildren treated for a skin disorder compared to their peers.

## Methods

### Databases

We linked Scotland-wide, individual-level data from four health databases, held by Public Health Scotland, and four education databases, held by the Scottish Exchange of Educational Data (ScotXed). The linkage methodology has been described previously [44–50]. The Prescribing Information System (PIS) collects information on all prescriptions dispensed to Scottish residents by community pharmacies or primary care. The Scottish Morbidity Record (SMR) 02 maternity database collects data on maternal, obstetric and child factors. SMR 01 and SMR 04 record acute and psychiatric hospital admissions, including dates of admission and discharge and International Classification of Diseases (ICD-10) diagnostic codes. The National Records of Scotland collect data from death certificates, including date and cause of death.

The pupil census is conducted annually by all local authority run primary, secondary and special schools across Scotland and includes whether a child has a special educational need and its type. Absences and exclusions are collected prospectively and appended at the end of the school year. The Scottish Qualifications Authority collects examination attainment data for all Scottish schoolchildren. The school leaver database collects information on pupils six months after leaving school: paid/voluntary employment, higher/further education, training or unemployment.

### Inclusion criteria, definitions and outcomes

Our cohort comprised school pupils included in the annual school censuses undertaken between 2009 and 2013 inclusive. The mean number of observed school years per pupil was 3.65 (range 1–5 years). We excluded school records where age was recorded as <4 years or >19 years. For multiple births involving offspring of the same sex, it is not possible to be certain that the correct child has been linked; therefore, inclusion was restricted to singleton children. In the absence of primary care data or a national diagnostic database to identify patients we used PIS data to ascertain children treated for a skin disorder defined as: receipt of one or more prescriptions per year for an emollient (British National Formulary (BNF) section 13.2.1), topical corticosteroid (BNF section 13.4), or preparations for eczema or psoriasis (BNF section 13.5). Children who did not receive any of these drugs were included in the peer group.

We studied five educational outcomes: (i) annual number of days absent, (ii) annual number of school exclusions for challenging/disruptive behaviour, (iii) annual record of special educational need, (iv) attainment in national examinations, and (v) unemployment after leaving school. The latter two outcomes were restricted to pupils who left school during the study period. Absence and exclusion data were only available for years 2009, 2010 and 2012. Special educational need is defined as being unable to benefit from school education without help beyond that normally given to schoolchildren of the same age. We included special educational need attributed to intellectual disabilities, learning difficulties, dyslexia, language or speech disorder, physical, motor or sensory impairment, autistic spectrum disorder, social, emotional and behavioural difficulties, physical health conditions, and mental health conditions. Children could have more than one type recorded. Academic achievement was derived using the total number of awards attained at each level of the Scottish Credit Qualifications Framework (SCQF) [51] and converted into an ordinal variable: low, basic, broad/general and high attainment. Destination six months after leaving school was collapsed into a dichotomous variable of education/employment/training or unemployment. We studied two health outcomes: all-cause hospital admission and all-cause mortality. Data on hospital admissions and deaths were available until September 2014; providing a mean follow-up period of 4.3 years (maximum 5 years).

We adjusted for several confounders. The pupil census provided children’s sex, age and ethnicity. Area socioeconomic deprivation was derived from postcode of residence using the Scottish Index of Multiple Deprivation (SIMD) 2012, and children were allocated to general population quintiles. SIMD is derived from 38 indicators across 7 domains (income, employment, health, housing, geographic access, crime and education, skills and training) using information collected by datazone of residence (median population 769). Retrospective linkage to SMR 02 provided maternal age at delivery, parity, maternal smoking, gestation at delivery, mode of delivery and 5-minute Apgar score. We derived sex-, gestation-specific birthweight centiles as a measure of intra-uterine growth. We have previously demonstrated that chronic conditions such as ADHD [45,46], epilepsy [45,47], diabetes [45,48], asthma [45,49], and depression [45,50] are independently associated with poorer educational outcomes and health outcomes. Therefore, to enable adjustment for these comorbid conditions, we used PIS data to identify children dispensed medication for diabetes (insulin), epilepsy (any drug from BNF section 4.8), ADHD (methylphenidate hydrochloride, dexamphetamine sulphate, atomoxetine or lisdexamfetamine dimesylate), or depression (tricyclic antidepressant, selective serotonin reuptake inhibitor, mirtazapine or venlafaxine) on at least one occasion over the school year and those dispensed medication for asthma (inhaled corticosteroid plus beta agonist) twice or more over one year.

### Statistical analyses

The characteristics of children treated for a skin disorder were compared with their peers using chi square tests for categorical data, and chi square tests for trend for ordinal data. Special educational need, absences and exclusions, recorded annually, were analysed as yearly outcomes using population-averaged generalised estimating equations (GEEs) [52] which adjust for correlations between observations relating to the same pupil across different census years. We used the user-written quasi-likelihood under the independence model criterion (QIC) statistic to compare different correlation structures. The structure with the lowest trace QIC was selected as most appropriate [53]. Number of days absent and number of school exclusions were modelled using longitudinal GEE analyses with a negative binomial distribution and log link function. Number of possible annual attendances was used as an offset variable to adjust for individual exposure time. Special educational need was modelled using GEE analyses with a binomial distribution and logit link. Logistic regression (generalised ordinal and binary) was used to model exam attainment and unemployment respectively whilst hospitalisation and mortality were modelled using Cox proportional hazard models. In the Cox models, children prescribed relevant skin disorder medication were followed from the date of their first prescription. Children who did not receive medication for a skin disorder were followed from the date of their first school census year. The proportional hazards assumption was tested formally using the estat phtest command within Stata and, where the assumption did not hold, Poisson piecewise regression models were used. Multivariable models were run univariately then adjusted for sociodemographic and maternity confounders. We also explored age, sex and deprivation as potential effect modifiers. We tested for statistical interactions and undertook sub-group analyses where these were significant at P<0.05.

Finally, to test for confounding due to comorbid conditions, we re-ran the models for all of the main outcomes adjusting for the presence of diabetes, asthma, epilepsy, ADHD and depression, in turn, before including all in the model. All statistical analyses were undertaken using Stata MP version 14.1

### Approvals

The study was approved by the NHS Scotland Public Benefit and Privacy Panel. A data processing agreement was drafted between Glasgow University and Public Health Scotland and a data sharing agreement between Glasgow University and ScotXed. The linked data extract was anonymised, then stored and analysed within the national safe haven.

### The role of the funding source

The sponsor and funders had no role in the design and conduct of the study; collection, management, analysis, and interpretation of the data; preparation, review or approval of the manuscript, or decision to submit the manuscript for publication.

## Results

Between 2009 and 2013, 766,244 singleton children attended Scottish schools. Overall, 130,087 (17.0%) children were treated for a skin disorder (Table 1). Children treated for a skin disorder were more likely to be female and Asian, and less likely to live in deprived areas. They were more likely to have required assisted or operative delivery and had lower five-minute Apgar scores. Their mothers were older, less likely to have smoked during pregnancy and were more likely to have been nulliparous. Compared to their peers, children treated for a skin disorder were also more likely to be on medication for diabetes (0.5% versus 0.4% of peers, p = 0.006), asthma (11.8% versus 4.8% of peers, p<0.001), epilepsy (1.0% versus 0.6% of peers, p<0.001), ADHD (1.0% versus 0.9% of peers, p = 0.007) and depression (1.0% versus 0.6% of peers, p<0.001) (Table 1).

**Table 1:** Sociodemographic characteristics of schoolchildren by the presence of a treated Skin Disorder (SD).

		No SD		SD		
		N = 636,157		N = 130,087		
		N	%	N	%	P value
**Sociodemographic factors**						
Sex						
	Male	328,975	51.7	61,315	47.1	<0.001 *
	Female	307,182	48.3	68,772	52.9	
	Missing	0		0		
Deprivation quintile						
	1 (most deprived)	145,343	22.9	28,699	22.1	<0.001 **
	2	127,911	20.1	25,721	19.8	
	3	122,767	19.3	25,065	19.3	
	4	123,784	19.5	25,575	19.7	
	5 (least deprived)	115,836	18.2	24,933	19.2	
	Missing	516		94		
Ethnic group						
	White	605,003	95.1	119,811	92.1	<0.001 *
	Asian	12,036	1.9	5,624	4.3	
	Black	1,222	0.2	682	0.5	
	Mixed	5,211	0.8	1,473	1.1	
	Other	1,691	0.3	396	0.3	
	Missing	10,994		2,101		
**Medication for comorbid conditions**						
	Diabetes	2,705	0.4	625	0.5	0.006 *
	Asthma	30,568	4.8	15,332	11.8	<0.001 *
	Epilepsy	4,054	0.6	1,260	1.0	<0.001 *
	ADHD	6,067	0.9	1,346	1.0	0.007 *
	Depression	4,088	0.6	1,254	1.0	<0.001 *
**Maternity factors**						
Maternal age (years)						
	≤24	176,254	27.7	33,624	25.8	<0.001 **
	25–29	186,894	29.4	37,646	28.9	
	30–34	178,753	28.1	38,182	29.4	
	≥35	94,244	14.8	20,635	15.9	
	Missing	12		0		
Maternal smoking						
	No	403,035	63.4	88,079	67.7	<0.001 *
	Yes	159,477	25.1	28,312	21.8	
	Missing	0		0		
Parity						
	0	282,925	44.7	62,740	48.5	<0.001 **
	1	220,095	34.8	44,047	34.1	
	>1	130,120	20.6	22,450	17.4	
	Missing	3,017		850		
Mode of delivery						
	SVD	431,855	67.9	84,364	64.9	<0.001 *
	Assisted vaginal	74,596	11.7	17,061	13.1	
	Breech vaginal	1,895	0.3	338	0.3	
	Elective CS	48,170	7.6	10,144	7.8	
	Emergency CS	79,500	12.5	18,156	14.0	
	Other	139	0.0	24	0.0	
	Missing	2		0		
Gestation (weeks)						
	<24	27	0.0	2	0.0	0.547 **
	24–27	944	0.1	181	0.1	
	28–32	5,880	0.9	1,178	0.9	
	33–36	29,471	4.6	6,131	4.7	
	37	31,315	4.9	6,304	4.8	
	38	79,519	12.5	16,473	12.7	
	39	131,617	20.7	27,123	20.9	
	40	191,706	30.2	38,724	29.8	
	41	141,803	22.3	29,390	22.6	
	42	22,741	3.6	4,383	3.4	
	43	523	0.1	107	0.1	
	>43	115	0.0	25	0.0	
	Missing	496		66		
Sex-gestation-specific birthweight centile						
	1–3	26,182	4.1	5,304	4.1	0.992 **
	4–10	56,999	9.0	11,647	9.0	
	11–20	75,639	11.9	15,709	12.1	
	21–80	373,841	58.8	76,280	58.7	
	81–90	54,248	8.5	11,115	8.6	
	91–97	34,274	5.4	6,947	5.3	
	98–100	14,131	2.2	2,948	2.3	
	Missing	843		137		
5-minute Apgar score						
	1–3	3,162	0.5	547	0.4	<0.001 **
	4–6	6,094	1.0	1,208	0.9	
	7–10	620,650	98.5	126,762	98.6	
	Missing	6,251		1,570		

SD Skin Disorder; N number; ADHD Attention Deficit Hyperactivity Disorder

SVD spontaneous vaginal delivery; CS Caesarean section

* chi^2^ test for association;

** chi^2^ test for trend

Analyses of school absences and exclusions were conducted on 702,210 children. Children treated for a skin disorder had more days absent from school than their peers after adjusting for sociodemographic and maternity factors (IRR 1.07, 95% CI 1.06–1.08, p<0.001). There was an interaction with age (p<0.001) whereby the association between skin disorder and absenteeism was stronger among children aged 11–14 years (IRR 1.09, 95% CI 1.07–1.11, p<0.001) compared to children older than 14 years (IRR 1.03, 95% CI 1.01–1.04, p<0.001) and younger than 11 years (IRR 1.07, 95% CI 1.06–1.08, p<0.001).

Treated skin disorders were associated with lower rates of school exclusion after adjusting for sociodemographic and maternity confounders (IRR 0.91, 95% CI 0.85–0.97, p = 0.004). However, on subgroup analyses, this association only reached statistical significance among boys (IRR 0.91, 95% CI 0.84–0.98, p = 0.015), and children aged >14 years (IRR 0.86, 95% CI 0.78–0.94, p = 0.001).

Children treated for a skin disorder were more likely to have a record of special educational need after adjusting for sociodemographic and maternity factors (OR 1.19, 95% CI 1.17–1.21, p<0.001). There was an interaction with age (p<0.001) whereby the association was stronger among children older than 14 years of age (OR 1.28, 95% CI 1.23–1.32, p<0.001) compared to children aged 11–14 years (OR 1.23, 95% CI 1.19–1.27, p<0.001) and children younger than 11 years (OR 1.13, 95% CI 1.10–1.16, p<0.001).

Among the 139,205 children who had undertaken exams, there was no significant association between treatment for a skin disorder and academic attainment after adjustment for sociodemographic and maternity factors (OR 1.01, 95% CI 0.96–1.05, p = 0.735). Among the 217,924 children who left school during the study period, 3,917 (25.5%) of those treated for a skin disorder left school before 16 years of age compared with 58,864 (29.1%) of their peers (p<0.001). Having a skin disorder was associated with lower risk of unemployment overall after adjusting for sociodemographic and maternity factors (OR 0.90, 95% CI 0.85–0.96, p<0.001). However, on subgroup analyses, this association was only statistically significant among boys (IRR 0.86, 95% CI 0.79–0.94, p = 0.001), and children in the most deprived quintile (IRR 0.83, 95% CI 0.74–0.92, p = 0.001).

**Fig 1:**
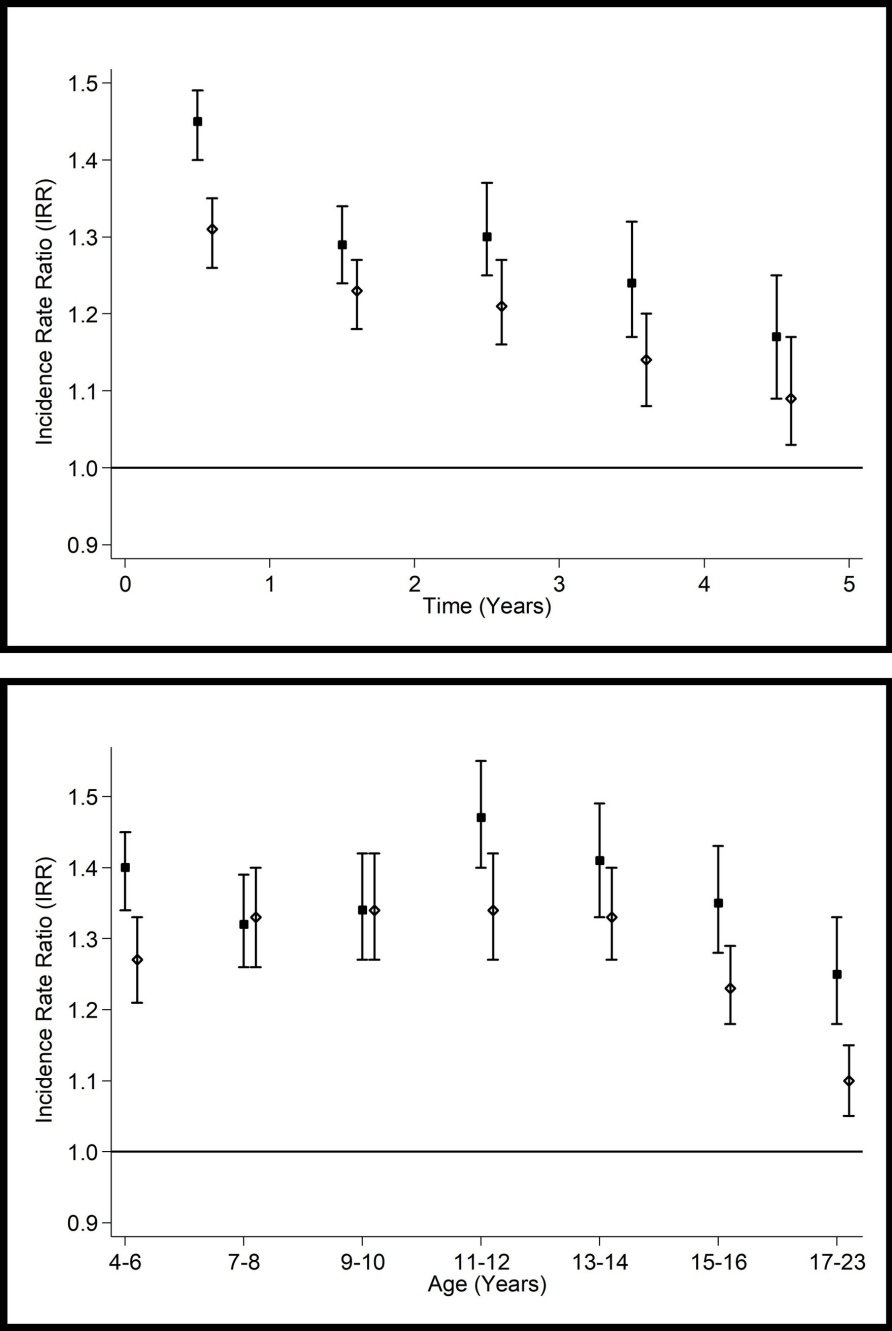
Association between treatment for a skin disorder and all-cause hospitalisation by sex (boys = solid square; girls = hollow diamond). a. By time from commencement of medication. b. By age at admission. Adjusted for age, sex, deprivation quintile, ethnic group, maternal age, maternal smoking, parity, mode of delivery, gestation at delivery, sex- gestation-specific birthweight centile and 5-minute Apgar score. SIMD–Scottish Index of Multiple Deprivation.

Linkage to hospital records provided 2.84 million person years of follow-up. The mean follow-up duration was 3.7 years. Of the 766,244 children, 153,002 were admitted to hospital at least once and 294,864 hospital admissions occurred in total. In the Cox proportional hazards models, children treated for a skin disorder experienced increased risk of hospitalisation for any cause (HR 1.29, 95% CI 1.27–1.30, p<0.001). However, the proportionality assumption was not met (p<0.001). On running a Poisson piecewise regression model, children treated for a skin disorder were more likely to be hospitalised over all five study years and across all ages. However, the incidence rate ratio was highest in the first recorded year of treatment (IRR 1.38, 95% CI 1.35–1.41, p<0.001) and fell over the remaining four years. It also peaked among children aged 11–12 years (IRR 1.40, 95% CI 1.35–1.46, p<0.001) on stratifying the analyses by age at admission (<6, 7–8, 9–10, 11–12, 13–14, 15–16, >17 years). There was an interaction with sex (p<0.001) whereby the association between a treated skin disorder and hospital admission was present in both boys and girls but stronger in the former. Fig 1A and 1B show fully adjusted incidence rate ratios, stratified by sex, for all-cause hospitalisation for each year of follow-up and by age at admission derived from Poisson piecewise regression models.

There were 491 deaths over the follow up period; 87 among children treated for a skin disorder and 404 among their peers. Children treated for a skin disorder were more likely to die from any cause over follow-up after adjusting for sociodemographic and maternity factors (HR 1.50, 95% CI 1.18–1.90, p<0.001). Among all children who died, the three most commonly recorded causes of death were external causes (37.5%), neoplasms (17.8%) and diseases of the nervous system (10.4%). External causes accounted for a smaller proportion of deaths among children treated for skin disorders than their peers (20.7% versus 41.1%) as did neoplasms (13.8% versus 17.8%). In contrast, children treated for a skin disorder were more likely to have their cause of death attributed to diseases of the nervous system (14.9% versus 9.4%).

Adjusting the main analyses for presence of comorbid conditions, both individually and then together, did not alter the findings (Table 2). After adjusting for all of the comorbid conditions, children treated for a skin disorder still had increased risk of absenteeism (IRR 1.05, 95% CI 1.04–1.05, p<0.001), special educational need (OR 1.14, 95% CI 1.12–1.16, p<0.001), hospitalisation (HR 1.22, 95% CI 1.22–1.24, p<0.001), and mortality (HR 1.31, 95% CI 1.03–1.67, p = 0.027), but decreased risk of school exclusion (IRR 0.91, 95% CI 0.86–0.97, p = 0.006) and unemployment (OR 0.89, 95% CI 0.84–0.95, p<0.001). There remained no association with academic attainment (OR 0.99, 95% CI 0.95–1.04, p = 0.762). Confirmation of these results will require further research, identifying patients by their diagnosis and recording the clinical severity of their condition.

**Table 2:** Educational and health outcomes additionally adjusted for comorbid chronic conditions.

	Absence		Exclusion		SEN		Attainment		Unemployment		Admission		Mortality	
	IRR	95% CI	IRR	95% CI	OR	95% CI	OR	95% CI	OR	95% CI	HR	95% CI	HR	95% CI
univariate	0.99	0.98–1.00	0.67	0.63–0.72	1.07	1.05–1.09	0.84	0.81–0.87	0.81	0.76–0.86	1.26	1.24–1.28	1.45	1.15–1.83
Multivariate 1 *	1.04	1.03–1.05	0.83	0.78–0.89	1.14	1.12–1.16	0.98	0.94–1.02	0.89	0.84–0.94	1.27	1.25–1.29	1.48	1.17–1.87
Multivariate 2 **	1.07	1.06–1.08	0.91	0.85–0.97	1.19	1.17–1.21	1.01	0.96–1.05	0.90	0.85–0.96	1.29	1.27–1.30	1.50	1.18–1.90
Multivariate 2 also adjusted for diabetes	1.07	1.06–1.08	0.91	0.85–0.97	1.19	1.17–1.21	1.01	0.96–1.05	0.90	0.85–0.96	1.28	1.27–1.30	1.50	1.18–1.90
Multivariate 2 also adjusted for asthma	1.05	1.04–1.06	0.92	0.86–0.98	1.16	1.14–1.19	1.00	0.96–1.04	0.90	0.85–0.96	1.23	1.22–1.25	1.44	1.13–1.83
Multivariate 2 also adjusted for epilepsy	1.07	1.06–1.08	0.91	0.85–0.97	1.17	1.15–1.20	1.01	0.96–1.05	0.90	0.85–0.96	1.28	1.26–1.29	1.37	1.08–1.73
Multivariate 2 also adjusted for ADHD	1.07	1.06–1.08	0.90	0.85–0.96	1.19	1.16–1.21	1.01	0.96–1.05	0.90	0.85–0.96	1.29	1.27–1.30	1.50	1.18–1.90
Multivariate 2 also adjusted for depression	1.07	1.06–1.07	0.91	0.85–0.97	1.18	1.16–1.21	1.00	0.96–1.05	0.90	0.84–0.95	1.28	1.26–1.30	1.47	1.16–1.87
Multivariate 2 adjusted for all comorbid conditions	1.05	1.04–1.05	0.91	0.86–0.97	1.14	1.12–1.16	0.99	0.95–1.04	0.89	0.84–0.95	1.22	1.20–1.24	1.31	1.03–1.67

*Adjusted for age, sex, deprivation quintile, ethnic group

*Also adjusted for maternal age, maternal smoking, parity, mode of delivery, gestation at delivery, sex- gestation-specific birthweight centile and 5-minute Apgar score

IRR Incidence Rate Ratio; OR Odds Ratio; HR Hazard Ratio; CI confidence interval; ADHD Attention Deficit Hyperactivity Disorder

## Discussion

This study is unique in identifying a large cohort of children from across Scotland who have received topical treatment for a skin problem. The absence of primary care data or a national database for outpatient diagnoses is a limitation and complicates the process of patient identification and deduction of type and severity of disorder. Nevertheless, in identifying patients by community prescriptions of topical treatment, this study found that, compared to peers, children treated for a skin disorder were more likely to be hospitalised, had more absences from school, were more likely to also be on medication for asthma, diabetes, epilepsy, depression and ADHD, and were more likely to be recorded as having special educational need. However, there was no evidence that this resulted in a longer-term adverse impact on attainment, which concurs with previous studies on atopic dermatitis [42,43], and employment. These latter findings may provide reassurance to affected children and their parents.

Whilst the findings relating to educational and employment outcomes were reassuring, we did demonstrate that, even after adjusting for comorbid conditions, children treated for a skin disorder were at increased risk of all-cause mortality. It is possible however, that patients with terminal disease, disease that results in immobility, or disease treatment that can result in skin side effects, may be prescribed emollients and steroids in the community for rashes that do not correspond to the innate, chronic skin disease that we would want to study. In any observational study, residual confounding and reverse causation are possible. Therefore, these findings require corroboration in other studies and if replicated merit further investigation. In particular, our findings that children with a skin disorder had less risk of exclusion, albeit only statistically significant among boys and those older than 14years of age, and less risk of unemployment, albeit only statistically significant among boys and those in the most deprived quintile, were unexpected and may be due to residual confounders. Therefore, these results should be corroborated and investigated further in future studies.

Previous studies investigating health outcomes of children with skin disorders are limited in number. Whilst adults with skin disorders reportedly suffer from increased mortality [35,36], studies reporting hospitalisation and mortality among children and adolescents are rare [37,38]. However, our finding of increased risk of death does concur with a previous study reporting greater premature mortality among children with psoriasis [38]. Our findings of increased special educational need and treatment for asthma, diabetes, epilepsy, depression and ADHD concur partially with previous studies which have reported greater risk of asthma [15,16,18] and ADHD [29–31] among children with eczema, increased incidence of diabetes [14,19,21] among children with psoriasis, and more psychiatric disorders and mental health problems among both including specifically depression and anxiety [22–28]. We have previously reported that children treated for diabetes, asthma, epilepsy, ADHD, and depression have poorer educational and health outcomes, including hospitalisation and mortality, compared to peers, and therefore adjusted for these conditions in our analyses [45–50]. However wider comorbidities exist which we could not adjust for. For example, childhood eczema has also been associated with comorbid allergic rhinitis, hay fever and autoimmune disorders [15–17] and even suicidal ideation/behaviour [32–34] whilst psoriasis has been associated with obesity, cardiovascular disorders, rheumatoid arthritis, and Crohn’s disease [14,19–21] as well as more psychiatric hospitalisations [37]. Therefore, in addition to the direct physical and mental effects associated with skin disorders, it is possible that the greater hospitalisation and mortality observed may be partly attributable to the increased physical and mental comorbidities associated with both eczema and psoriasis over and above those adjusted for in our analyses.

Previous studies investigating educational outcomes of children with skin disorders are rarer still and face methodological limitations. Indeed, a systematic review of educational and employment outcomes among children with eczema identified only one eligible study [41]. General school difficulty among affected children has been reported previously [40] and reports of increased absenteeism [39] yet unaffected attainment [42,43] concur with our own findings. However, the latter studies on attainment were limited whereby one only investigated attainment on entry to school at age 11 for children with eczema, rather than final attainment on leaving school, and the other only investigated attainment among boys with severe atopic dermatitis limiting generalisability. We have previously demonstrated that children treated for diabetes, asthma, epilepsy ADHD, and depression have greater risk of absenteeism and special educational need compared to peers however our new findings pertaining to skin disorders remained after adjusting for presence of those conditions [45–50]. In addition to the direct effects of having a skin disorder, our findings of increased absenteeism and special educational need may therefore similarly be partly attributable to the wider increase in physical and mental comorbidities experienced by affected children which we could not adjust for as highlighted already.

Our study addressed a significant gap in the literature by investigating several health and educational outcomes. Ours was a large, non-selective study including children attending schools across the whole of Scotland. Because the sampling frame was mainstream and special schools, rather than hospital clinics, ascertainment of children treated for a skin disorder was not restricted to the most severe skin disorders and we were able to adjust for a wide range of potential confounders: sociodemographic, maternity and comorbid conditions. The large cohort provided sufficient power to test for statistical interactions and undertake sub-group analyses where appropriate, and we were able to analyse a wide range of educational, employment and health outcomes within the same cohort of children.

Our study only included children attending local authority maintained schools; however, in Scotland, less than 5% of children attend private schools. According to the 2011 Scottish Census, 11% of Scottish residents aged 5–19 years were born outside of Scotland; consistent with the 12% of Scottish children we could not link to Scottish maternity records. We used existing, administrative databases established for other purposes. However, they undergo regular quality assurance checks. Linkage of education and health records relied on probabilistic matching which has been validated to be 99% accurate for singletons [44].

Our findings require corroboration in other studies and if replicated merit further investigation. Whilst acknowledging these uncertainties we conclude that, based on the observed poorer health outcomes, the introduction of treatment plans is welcome as an attempt to provide children and adolescents who have skin disorders with more personalised healthcare. Given that these children regularly experience comorbid physical and mental health conditions, a more joined up approach to care is required. Indeed, in light of the common psychological burden of these conditions, a recent study highlighted unmet psychological and health care needs of adolescents transitioning from paediatric to adult services and a requirement for more dedicated dermatology clinics in the UK with embedded psychological support capable of providing developmentally appropriate healthcare and psychosocial support for this population [54]. Having observed poorer attendance and greater special educational need among affected children we conclude that early identification and support should be a priority and that interventions should focus on reducing the risk of school absenteeism. In order to reduce school absenteeism or mitigate its effects, children treated for skin disorders should receive integrated care from a multidisciplinary team covering physicians, teachers, parents, and where mental health comorbidities exist, educational psychologists and social services as appropriate. Their management should extend beyond healthcare to a programme of school-based interventions.

## Conclusion

Children prescribed topical treatment for a skin disorder have an increased risk of hospitalisation and mortality, miss more days of school, have greater special educational need than peers, and are more likely to be treated for other chronic conditions including diabetes, asthma, epilepsy, ADHD and depression. However, there was no evidence of an adverse impact on longer-term educational outcomes in terms of poorer exam grades whilst at school and unemployment after leaving school. Whilst our findings relating to educational and employment outcomes are reassuring, the association with increased risk of mortality is alarming and merits further investigation. The introduction of treatment plans is welcome as an attempt to provide children who have skin disorders with more personalised and joined-up healthcare. School interventions should include measures aimed at reducing absenteeism and providing support in the form of integrated care from a multidisciplinary team.

## Supporting information

S1 Checklist STROBE statement—checklist of items that should be included in reports of observational studies. (DOCX)
